# Implementation, barriers, and recommendations for further development of advance care planning for the last phase of life in nursing homes in Germany (Gut-Leben): protocol for a mixed-methods study

**DOI:** 10.1186/s12904-023-01147-y

**Published:** 2023-03-24

**Authors:** Stephanie Stiel, Anna Levke Brütt, Jona T Stahmeyer, Anne W E Bockelmann, Tanja Schleef, Anna Völkel, Falk Hoffmann

**Affiliations:** 1 Institute for General Practice and Palliative Care, Hannover Medical School, Carl-Neuberg-Straße 1, 30625 Hannover, Germany; 2 Junior Research Group for Rehabilitation Sciences, Department of Health Services Research, Carl von Ossietzky University Oldenburg, Oldenburg, Germany; 3 Health Services Research Unit, AOK Niedersachsen, Hannover, Germany; 4 Division of Outpatient Care and Pharmacoepidemiology, Department of Health Services Research, Carl von Ossietzky University Oldenburg, Oldenburg, Germany

**Keywords:** End of life care, Advance care planning, Nursing home

## Abstract

**Background:**

Nursing home entry often marks the transition into the end-of-life. In 2018, Germany legally introduced reimbursement for advance care planning (ACP according to § 132 g SGB V) in nursing homes that applied for ACP approval to improve end-of-life care. The Gut-Leben project aims to evaluate the implementation and barriers of ACP in nursing homes in Germany, with a special focus on the federal state of Lower Saxony, and provide practical recommendations for further development of end-of-life care.

**Methods:**

This mixed-methods study spreads across five work packages (WP) over a three-year period. WP 1 will explore the approval process, implementation, and barriers to ACP in nursing homes. Data will be collected through a national postal survey in a random sample of *n* = 600. WP 2 will explore whether documented ACP reports are applicable as a data source for research (expecting up to 500 available ACP reports). In WP 3a and 3b, data on the ACP process will be collected in *n* = 15 approved nursing homes in Lower Saxony over a 12-months period. For WP 3a and WP 3b, data on ACP conversations (*n* = 600) and on end-of-life care paths (*n* = 300) will be collected by facilitators and nurses of the respective nursing homes. In WP 4, residents’ characteristics upon entry and changes in these characteristics over the length of stay are analyzed, utilizing claims data from the AOK Lower Saxony (expected sample of about 100,000 people entering nursing homes over a 10-years period). WP 5 connects, interprets, and reflects on the findings from WP 1–4 through focus groups and individual interviews with facilitators, nursing staff, residents, relatives, and care providers. Within a participatory approach, a practice advisory board will be set up existing of personal of nursing homes and will be closely involved in the whole research process.

**Discussion:**

In summary, the Gut-Leben project provides insight into the implementation and barriers of ACP in nursing homes according to German legislation for the first time, including practitioners’ and residents’ perspectives. Insights will help the further development of ACP in Germany through practical recommendations based on quantitative and qualitative data.

## Background

### The nursing home as a place of care for people at the end of life

Currently, around 820.000 people in Germany live in nursing homes [1]. This number has increased in recent years and will increase further due to demographic changes [2]. Entry into a nursing home usually marks the transition into the end-of-life phase [3], indicating the importance of the nursing home setting for end-of-life care [4]. According to an own study based on routine data of a large German health insurance fund, the median time between nursing home entry and death is 706 days [5, 6]. However, the variance was large, and the mortality peaked during the first six months after nursing home entry, which was also found in the international literature [7]. A Swedish study showed that between 2002 and 2012, the time between nursing home entry and death became even shorter [8]. Furthermore, in a local study in the region of Hannover, the proportion of the population dying in nursing homes has increased from 15.3% to 2007 to 27.1% in 2017, indicating a decrease in the duration of stay [9].

People at the end-of-life are especially vulnerable to both undertreatment (e.g., inadequate pain therapy, and no or delayed inclusion in palliative care) and overtreatment (e.g., due to not or no longer indicated diagnostic or therapeutic measures) [10]. The issue of overtreatment is especially relevant for the nursing home setting, where inappropriate hospitalizations are a known issue. In the literature, short unplanned hospital stays at the end of life, especially in case of present dementia, are often deemed inappropriate [11]. Furthermore, hospital stays at the end of life often do not reflect residents’ personal will and care preferences [12].

Nevertheless, hospitalization rates are high for nursing home residents in Germany, especially in the last weeks before death [5]. In a systematic review of 35 studies, we found that internationally a median of 22.7% of nursing home residents died in a hospital, and 33.2% were hospitalized in the last month of life [13]. In Germany, these proportions were higher, 29.5% of nursing home residents died in a hospital, and 51.5% were hospitalized in the last month of life [5, 6]. Furthermore, international figures show notably lower proportions of hospitalization for nursing home residents with dementia at the end of life [14], while in Germany, no differences were found [5, 6]. In Germany, hospital transports are often done for legal considerations [15]. Furthermore, nursing home residents’ will and care preferences are often unknown [16, 17].

### The implementation of the concept of advanced care planning in Germany

To improve end-of-life care, the concept of “Advance Care Planning” (ACP) is often implemented, especially in Anglo-American countries [18]. ACP is a structured and continuous process in which trained facilitators document the medical and care preferences for the future, together with patients, relatives, and others involved in the care process [19]. Thereby ACP allows for care preferences to be followed in situations where patients cannot make decisions on or communicate their preferences [20]. Furthermore, the inclusion of relatives in the ACP process is important because knowing the patients’ prognosis results in a higher preference for palliative care and a lower likelihood of stressful end-of-life measures (such as placing a feeding tube, parenteral fluids, or hospital transports), as well as an increased satisfaction of the relatives themselves [21, 22]. In countries where ACP is more widely applied, hospitalization rates of nursing home residents at the end of life are comparatively lower [13].

In 2018, Germany introduced a new legal framework in § 132 g Book V of the German Social Code (SGB V) (Healthcare planning for the last phase of life), which enabled nursing homes to reimburse costs for services based on the concept of ACP with health insurance providers. To be approved, nursing homes must employ at least one ACP facilitator trained in theoretical and practical content. In addition, nursing homes must provide information on the organization of ACP and the embedding in the overall structure, including internal and external cooperation. ACP services can be provided individually or in collaboration with other nursing homes. The compensation is issued in a lump sum, which finances 1/8 of the facilitator’s position per 50 insured residents per facility.

The facilitators should make a voluntary offer to discuss the residents’ wishes, compose appropriate care documents for the end-of-life and ensure their availability. The factual content of the ACP process is left to the nursing homes. Topics of ACP conversations can be, for example, expectations of medical and nursing care processes, such as possibilities, intensity, and extent of medical interventions, handling of emergencies, and palliative and psychosocial care offers at the end-of-life. Relatives, caregivers, and care providers can be included in this process. The ascertained preferences are recorded in resident care documents (especially the living will, power of attorney, and appointment of legal representatives) to create legal certainty inside and outside the nursing home. When completing an initial or revised ACP process, it must be reported to the resident’s health insurance, although it is not relevant to remuneration.

### Objective and research questions

To date, no structured and published data regarding the implementation and organization of ACP in German nursing homes are available. It is unknown to what extent ACP has been implemented so far and what the barriers and context factors are.

Therefore, the Gut-Leben project aims to give a comprehensive picture of the possibilities created by § 132 g SGB V for implementing ACP in German nursing homes. Possible barriers to the implementation will be identified, ACP processes will be examined in more detail, and relevant contextual factors will be determined at the level of nursing homes and residents. Finally, we will make recommendations to develop the legal framework further. To ensure a close connection between research and the real-life care setting, a practice advisory board will be implemented.

The following research questions will be answered:

- What are possible barriers to the approval of German nursing homes to provide ACP (according to § 132 g SGB V)?
- Are the legally required reports about the counseling process in nursing homes applicable for research?
- How is the ACP process implemented and organized in approved nursing homes? What do end-of-life care paths look like in these nursing homes?
- What characterizes nursing home residents (regarding factors relevant for further development of ACP) upon entry? Did these characteristics change over recent years?
- How do stakeholders from approved nursing homes interpret the projects’ findings? How can these findings affect the further development of ACP in German nursing homes?

## Methods and design

**Fig. 1:**
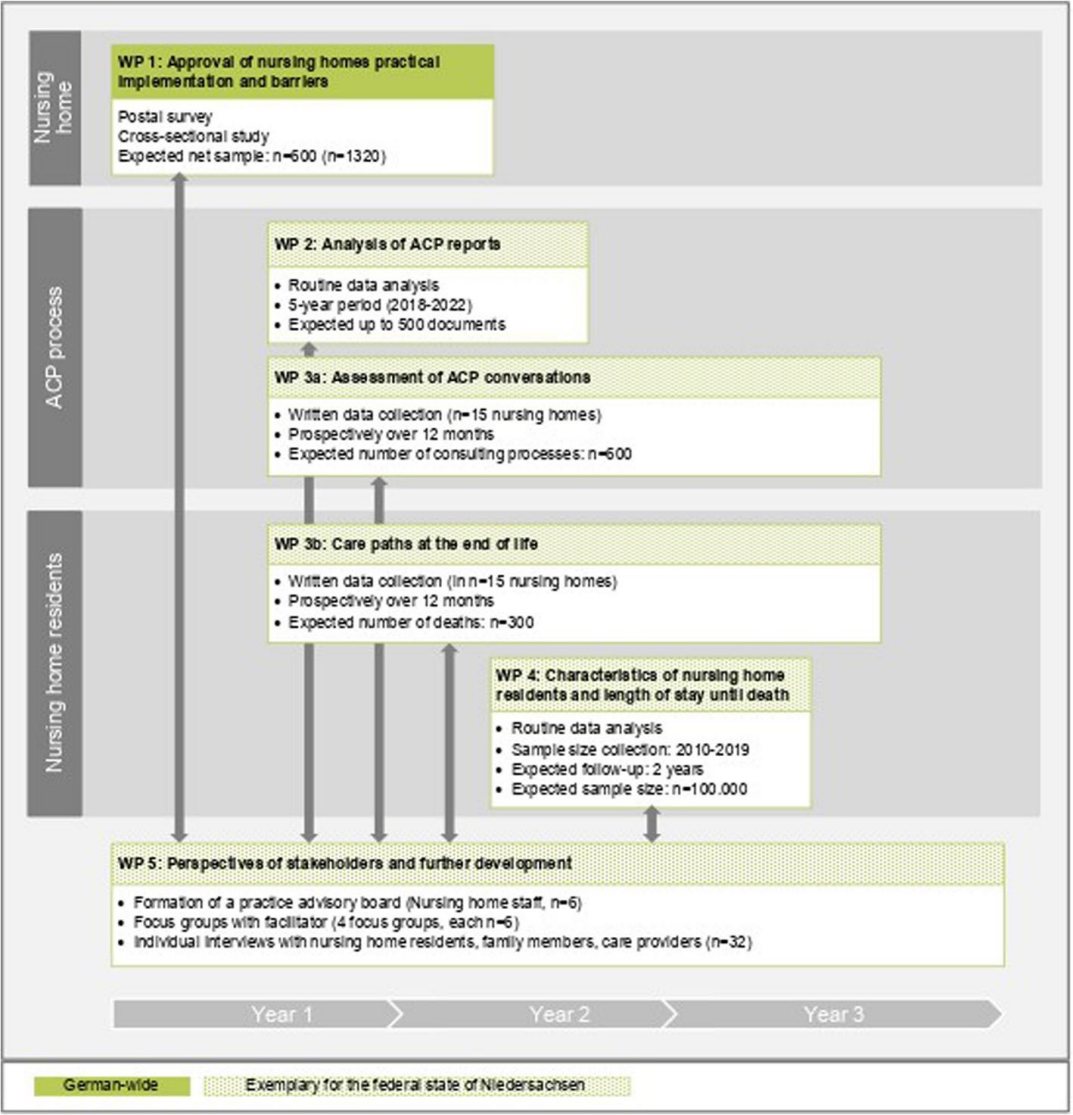
Timeline, integration, and short methods of the WP`s of the Gut-Leben project

We describe the protocol of a mixed-methods study involving qualitative and quantitative parts with participatory aspects (Gut-Leben). The project spreads across five work packages (WP) over a three-year period. Whereas WP 1 will include a national representative sample, all further WP will focus on Lower Saxony (one of 16 German federal states, including about 8 million inhabitants, about 9.8% of the German population). Figure 1 shows the timeline of the Gut-Leben project and the integration of the different WPs with a short summary of the methods planned.

### WP 1: approval of nursing homes, practical implementation, and barriers

In WP 1, we will explore the approval process, implementation, and perceived barriers to ACP in German nursing homes. A cross-sectional study will be conducted as a nationally representative postal survey aimed at the management of nursing homes. The main topics of the survey are to what extent legal possibilities of ACP in German nursing homes are known, how far approval exists or has been requested (individually or in cooperation) by nursing homes, and the experienced barriers in (not) doing so. Furthermore, structural data on nursing homes will be collected (e.g., number of residents, employee pool, qualifications, and the number of general practitioners involved in the care), as well as access to and cooperation with regional palliative and psychosocial support providers. For approved nursing homes, data on the structure of the ACP offer will be collected (e.g., number of ACP facilitators, cooperation with other nursing homes, the structure of ACP conversations, and how long the nursing homes have been approved). For unapproved nursing homes, data will be gathered on which conditions have already been fulfilled for approval and which offers related to ACP, and palliative care exists. Before the survey roll-out, a pre-test will be conducted in multiple nursing homes.

Assuming a proportion of 50% with a 95% confidence interval and precision of ± 4% (46–54%), a net sample of 600 responding nursing homes is required (sample calculation carried out with OpenEpi version 3.1). This sample will include a sufficient number of approved (expected *n* = 60) and unapproved nursing homes (expected *n* = 540). Assuming a response of 45.5%, as reached by our previous survey in nursing homes [23], a sample of 1,320 will be required. A simple random sample will be drawn from the nursing home addresses in the AOK-Pflegenavigator (about 11,500 nursing homes). The following measures to increase the response rate will be implemented: (1) a piloted survey of max. four pages with a cover letter addressed personally to the nursing home manager, and (2) a reminder about three weeks later to the whole sample, including another copy of the questionnaire [24].

The gathered data is transferred to IBM SPSS (IBM Corporation, Armonk, NY, USA) and thoroughly checked for plausibility. Additionally, a non-response analysis will be conducted, exploring regional differences in response. The collected quantitative data will be analyzed with descriptive statistical methods.

This WP sheds light on the extent to which nursing homes are familiar with ACP according to § 132 g SGB V and the practical barriers to approval. We will be able to describe what characterizes approved nursing homes and the existing offers for care planning and palliative care offered by unapproved nursing homes.

### WP 2: Analysis of ACP reports

For WP 2, we will cooperate with the AOK Lower Saxony to study whether the documented paper and electronic reports (according to annex 2 of § 132 g Abs. 3 SGB V) are applicable for research purposes. We will assess, e.g., how often reports are filed by how many nursing homes, how often these reports are paper-based or electronic, how often fields are not or not plausibly filled in, and how often free-text answers were given. At the time of the execution of this WP, five years of possible reports should be available, and the quantity and quality of available reports over the years will also be evaluated. We expect up to 500 available reports. In case the number of reports greatly exceeds this number, an algorithm will be established, with which a random sample will be drawn.

The exact size of the analysis will be first known after the reports’ systematic inspection and plausibility checks, for which an appropriate data mask will be developed. Then, the first analyses will be conducted as far as the data allows. Questions include how often initial and renewed facilitator processes are carried out, how many facilitators nursing homes employ, and how many conversations they conduct.

This WP will provide information on the extent to which the legally required reports are available and useable for research purposes, what information they contain, and to what extent initial framework data on the ACP processes can be obtained.

### WP 3a & 3b: Assessment of ACP conversations and care paths at the end of life

In WP 3a, information on the facilitator-led ACP conversation will be prospectively collected over 12 months from ACP-approved nursing homes in Lower Saxony. Data on the contents and the arrangement of each conducted ACP conversation, as well as their embedding in the ACP process, will be collected, such as discussed themes and contents, use of support materials, outcomes of the conversations, preparation, and actualization of ACP reports.

Furthermore, framework data on the ACP conversations (e.g., duration, amount of follow-up conversations, the inclusion of relatives and care providers, and atmosphere) and characteristics of the residents (e.g., age, sex, dementia, date of nursing home entry), duration of the conversation, amount of follow-up conversations, the inclusion of relatives and care providers, and atmosphere will be collected.

Due to the explorative character of the study, no sample size calculation will be conducted. Assuming 90 residents per nursing home [23], of which 80 are allowed to be publicly ensured, we estimate that an average of 40 ACP conversations take place each year, per nursing home (multiple conversations per year per resident are possible), with a convenience sample size of 15 approved nursing homes, we expect to collect data from 600 ACP conversations. The recruitment of nursing homes will take place by post and telephone through the available list of approved nursing homes from the AOK Lower Saxony (ca. *n* = 200 in Lower Saxony).

WP 3b will provide insight into characteristics of care paths at the end of life in approved nursing homes. The data collection will occur alongside WP 3a in the same sample of approved nursing homes. Data will be prospectively collected over 12 months for all residents that die in this period. Collected data for each deceased resident includes, i.e., information on hospitalization, place of death, involvement of specialized ambulatory palliative care (SAPV) and other palliative structures, and nutritional support. Furthermore, data on the residents’ individual ACP processes, such as those collected in WP 3a, will be gathered. Based on an average of 90 residents per nursing home [23] and a mortality rate of 38 per 100 person-years after nursing home entry [5], which will be lower for prevalent residents, we will assume 20 deaths per year per nursing home (*n* = 300 in total).

For WP 3a and 3b, specific, structured paper-based documentation forms will be developed by the practice advisory board, with the involvement of the approved nursing homes. To increase feasibility, they should be concise and take a maximum of 10 min to fill out. The documentation forms will be piloted in at least one nursing home. After receiving formal training, facilitators and nurses will collect data from the respective nursing home for WP 3a and 3b. No involvement of the residents is necessary, which increases the external validity of the WP. Complete documentation will be encouraged by a compensation of 10€ per case and regular personal or telephone contact.

The data gathered in WP 3a and 3b will be transferred to SPSS and thoroughly checked for plausibility. The analyses will be cluster adjusted to control for differences within and between nursing homes and take the data structure into account. However, differences between nursing homes regarding ACP conversations (WP 3a), the end-of-life care paths (WP 3b), and the number of ACP conversations per nursing home per year (WP 3a) will be explored.

Through the closely related WPs 3a and 3b, information on the practical implementation of ACP, its process, and whether preferences for end-of-life care are followed in approved nursing homes will be provided.

### WP 4: Characteristics of nursing home residents and length of stay till death

In WP 4, it will be examined what characterizes residents at nursing home entry and how they have changed over recent years. A cohort study (expected follow-up of two years) will be conducted using routine data from the AOK Lower Saxony of individuals of a minimum age of 65 + years who first entered a nursing home between 2010 and 2019. The AOK Lower Saxony ensures 2.9 million individuals, which translates to roughly one-third of all nursing home residents in Lower Saxony. Based on previous studies [23], we assume about 100,000 cases between 2010 and 2019. To be included, individuals have had to be insured at the AOK Lower Saxony for at least 365 days upon nursing home entry.

Follow-up will be up till death, end of the insurance period, or time of data extraction (expected is a follow-up of 2 years). Characteristics of the nursing home residents on entry will be analyzed, e.g., age, sex, care grade, dementia (as operationalized using ICD-10 codes from outpatient care in the quarter of nursing home entry [23]), and direct transfer from a hospital or short-term care to the nursing home. Furthermore, survival times after nursing home entry will be estimated using the Kaplan-Meier method. All analyses will be stratified by year of nursing home entry, sex, age group, and dementia. Based on a Swedish study by Schön et al. [8], we will also determine the percentiles of times to death (e.g., 10th, 25th, and 50th percentile) of residents by years, as well as the proportions of death residents in periods of 15, 30, 60, 90 and 180 days after nursing home entry. Moreover, multivariate analyses will be conducted to control for changes in baseline characteristics of nursing home residents over recent years (changes in age distribution, sex, dementia, or care grade).

The knowledge obtained through this WP about newly admitted nursing home residents will contribute to the further development of care offers and ACP in German nursing homes based on empirical data on the reality of care in nursing homes.

### WP 5: Perspectives of stakeholders and further development of ACP in german nursing homes

**Fig. 2:**
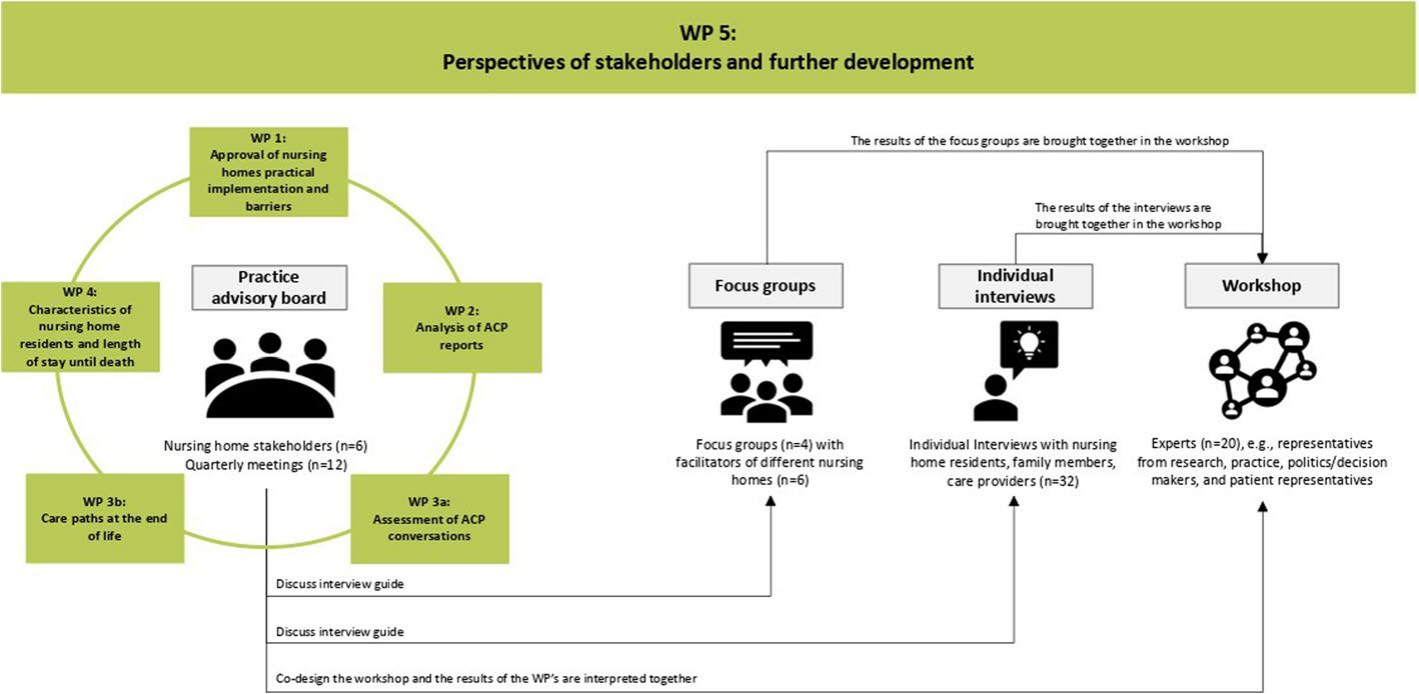
Workflow in WP 5

In this project-overreaching WP, the foundations for conducting the whole research process are laid, and findings from WP 1 to 4 are brought together, reflected on, and interpreted (see Fig. 2). Therefore, focus groups and individual interviews will be conducted to collect the subjective views of facilitators, residents, relatives, close confidants, and care providers to complete these data.

Additionally, a practice advisory board, comprising of personal of six nursing homes from approved nursing homes in Lower Saxony, will be established. The practice advisory board will be actively involved from the beginning in the project and the research process through quarterly face-to-face or video conferences. Recruitment channels and processes, study information and instruments, as well as outcomes will be presented and discussed. In this way experiences and assessments, as well as perspectives of stakeholders from the nursing homes in the research project will be accounted for. Furthermore, the board members can serve as multipliers and support the recruitment of focus group and interview participants.

Two researchers moderate the practice advisory board meetings and focus groups. The focus groups are orientated according to the guidelines discussed in the preceding practice advisory board meeting. In the focus groups, facilitators are first asked to introduce the reality of the implementation of ACP in their nursing home, then barriers and success factors are discussed.

Over the course of the project, residents, close confidants, and care providers will be individually interviewed (*n* = 32). In the problem-centered, semi-structured interviews, participants will give insight into the planning of the care process from their perspective. The corresponding topics, key questions, and their formulation will be discussed with the practice advisory board. One pilot interview with a resident, relative, and care provider will be conducted. In the individual interviews, we aim to gain insight into the implementation of ACP in nursing homes (e.g., acceptance, barriers, and support needs), as well as the arrangement of ACP conversations (e.g., time of initiation, unique features in case of dementia and additional qualifications required) from different perspectives.

With the participants’ consent, focus groups and individual interviews will be recorded on audio tape and then transcribed anonymously. The transcripts’ contents are evaluated according to Mayring [25]. Finally, a workshop will take place (*n* = 20 experts, e.g., representatives from research, practice, politics/decision makers, and patient representatives), in which the various results of the WP’s are brought together and interpreted in cooperation with the practice advisory board. In this workshop, strategies for further development of ACP in German nursing homes will be developed.

## Discussion

The Gut-Leben project is the first study to gather information on the implementation and possible barriers of ACP in German nursing homes according to § 132 g SGB V, as introduced in 2018. Including a strong participatory approach, the Gut-Leben project aims to provide practice-oriented recommendations for further development of ACP in German nursing homes. These recommendations will consider the contents of education and qualifications of the facilitators and nursing staff in homes and the design of additional ACP offers. Furthermore, a set of variables important for future electronic recording of the services provided during the ACP process can be developed. Our results can form a basis for structural changes or adjustments to the legal framework of ACP in German nursing homes. In addition, the project can contribute to more efficient use of resources and, at the same time, contribute to a higher quality of life, respectively end-of-life care, according to individuals’ preferences. Furthermore, the outcomes of the Gut-Leben project can also be applied to further extent § 132 g SGB V to include care-dependent individuals residing outside of the nursing homes, at home, or in other living arrangements.

### Study risks

During the planning, substantial risks and problems that could hinder the project were considered, and appropriate countermeasures were taken. The most impactful risks are presented below:

- Corona pandemic and associated measures

The persistence of the COVID-19 pandemic and corresponding measures, especially in the nursing home setting, cannot be ruled out. However, this does not significantly endanger the project, as there will be no direct recruitment of nursing home residents in WP 1–4. Data collection takes place through employees of the nursing homes and mainly through postal surveys or existing routine data. In WP 3a and 3b only the recruitment of the nursing homes themselves would require personal contact. WP 5 requires the recruitment of nursing home staff for the practice advisory board, ACP facilitators for the focus group, and residents, close confidants, and care providers for the interviews. However, recruitment and execution of these qualitative activities could also be realized through telephone or video conference.

b) Recruitment challenges

Problems with recruiting nursing homes for WP 3a and 3b could endanger the project’s success. However, at the time of planning the study and writing the proposal (February 2021), only 110 nursing homes offer ACP according to § 132 g Abs. 3 SGB V in Lower Saxony, and July 2022, this number increased to about 200. Accordingly, the number of approved nursing homes might be even larger when the respective WP begins. The planned sample of 15 approved nursing homes should thus not pose an issue, especially as there should be an intrinsic interest in the topic in eligible homes.

Moreover, nursing homes are included in the research process through the practice advisory board, which enables the discussion of timely strategies for recruitment for WP 3a and 3b. Through the two locations of the project in the North-west and the South of Lower Saxony, personal contact can quickly be established over the entire state. If needed, the state of Bremen can be included in the recruitment.

Recruitment problems for WP 5 on the part of the nursing home residents, or their non-participation, which could lead to selection bias, will be minimized through monetary compensation. Monetary compensation is also provided to ensure recruitment in several WPs.

iii) Incomplete documentation of ACP reports in the nursing homes

If the documentation, as required in WP 3a and 3b, is incomplete, this could pose a problem. Similar to two previous studies, IMREN [26] and HOMERN [16], incomplete documentation will be prevented by appointing staff responsible for the documentation and providing monetary compensation per filled-out questionnaire as an incentive. The questionnaire will be kept short and will be based on as much available information as possible (e.g., routine nursing documentation). Moreover, the paper-based documentation forms will be piloted and developed in cooperation with the practice advisory board. Additionally, there will be regular telephone or personal contact between the project staff and responsible staff at the nursing homes to discuss any issues.

iv) Legal changes

It is possible that the framework conditions for § 132 g Abs. 3 SGB V will be adjusted over the course of the study. So far, however, we are not aware of any corresponding proposals or plans as this legal framework was only introduced in 2018. If significant adjustments to the framework are implemented, the project can react to this by examining the implications of these changes as far as possible.

In summary, the Gut-Leben project will help the further development of ACP in German nursing homes through evaluation of the implementation of § 132 g SGB V, practical recommendations will be formulated based on quantitative and qualitative data gathered on the ACP process and end-of-life care paths in nursing homes. As the project includes a strong participatory approach, the recommendations provided will help to improve and further develop end-of-life care both inside and outside of nursing homes in Germany.

## Data Availability

Data sharing is not applicable to this article as no datasets have yet been generated or analyzed during the current study.

## Abbreviations

ACP: Advance Care Planning
WP: Work Package

## Declarations

All participants fill in and sign a consent to participate statement before study participation.
